# Treatment of severe pneumonia by hinokitiol in a murine antimicrobial-resistant pneumococcal pneumonia model

**DOI:** 10.1371/journal.pone.0240329

**Published:** 2020-10-15

**Authors:** Toshihito Isono, Hisanori Domon, Kosuke Nagai, Tomoki Maekawa, Hikaru Tamura, Takumi Hiyoshi, Katsunori Yanagihara, Eiji Kunitomo, Shoji Takenaka, Yuichiro Noiri, Yutaka Terao

**Affiliations:** 1 Division of Microbiology and Infectious Diseases, Niigata University Graduate School of Medical and Dental Sciences, Niigata, Japan; 2 Research Center for Advanced Oral Science, Niigata University Graduate School of Medical and Dental Sciences, Niigata, Japan; 3 Division of Periodontology, Niigata University Graduate School of Medical and Dental Sciences, Niigata, Japan; 4 Department of Laboratory Medicine, Nagasaki University Graduate School of Biomedical Sciences, Nagasaki, Japan; 5 Central Research and Development Laboratory, Kobayashi Pharmaceutical Co., Ltd., Osaka, Japan; 6 Division of Cariology, Operative Dentistry and Endodontics, Faculty of Dentistry & Graduate School of Medical and Dental Sciences, Niigata University, Niigata, Japan; University of Pittsburgh, UNITED STATES

## Abstract

*Streptococcus pneumoniae* is often isolated from patients with community-acquired pneumonia. Antibiotics are the primary line of treatment for pneumococcal pneumonia; however, rising antimicrobial resistance is becoming more prevalent. Hinokitiol, which is isolated from trees in the cypress family, has been demonstrated to exert antibacterial activity against *S*. *pneumoniae in vitro* regardless of antimicrobial resistance. In this study, the efficacy of hinokitiol was investigated in a mouse pneumonia model. Male 8-week-old BALB/c mice were intratracheally infected with *S*. *pneumoniae* strains D39 (antimicrobial susceptible) and NU4471 (macrolide resistant). After 1 h, hinokitiol was injected via the tracheal route. Hinokitiol significantly decreased the number of *S*. *pneumoniae* in the bronchoalveolar lavage fluid (BALF) and the concentration of pneumococcal DNA in the serum, regardless of whether bacteria were resistant or susceptible to macrolides. In addition, hinokitiol decreased the infiltration of neutrophils in the lungs, as well as the concentration of inflammatory cytokines in the BALF and serum. Repeated hinokitiol injection at 18 h intervals showed downward trend in the number of *S*. *pneumoniae* in the BALF and the concentration of *S*. *pneumoniae* DNA in the serum with the number of hinokitiol administrations. These findings suggest that hinokitiol reduced bacterial load and suppressed excessive host immune response in the pneumonia mouse model. Accordingly, hinokitiol warrants further exploration as a potential candidate for the treatment of pneumococcal pneumonia.

## Introduction

Pneumonia represents a major threat in a globalized society as it spreads easily by droplets. There are very few therapeutic agents that can tackle infection by drug-resistant bacteria or emerging viruses [1]. Furthermore, self-organ damage owing to excessive host immunity can also occur in severe pneumonia [2]. Therefore, treatment of severe pneumonia requires both elimination of drug-resistant microorganisms and suppression of hyperimmunity that damage the host lungs.

Hinokitiol, a tropolone-related compound has been isolated from *Chamaecyparis obtusa* and *Thuja plicata* Donn ex D. Don, which are trees in the cypress family, as β-thujaplicin [3, 4]. Previous studies have shown antitumor, anti-inflammatory, antioxidant, and antibacterial activities of hinokitiol [5–9]. We recently showed that hinokitiol was effective against *Streptococcus pneumoniae in vitro*, regardless of antimicrobial resistance [10]. At present, no studies have examined the effect of hinokitiol on pneumococcal infection *in vivo*.

*S*. *pneumoniae* is a gram-positive diplococcus resident in the human upper respiratory tract [11]. This bacterium causes not only pneumonia but also otitis media, sinusitis, bronchitis, and empyema [12], as well as invasive pneumococcal diseases, including meningitis, bacteremia, and septicemia [13]. The primary treatment of pneumococcal diseases relies on the use of effective antibiotics. In the early 2000s, macrolides were strongly recommended for pneumococcal pneumonia [14–16]; however, their widespread use has augmented the risk of infection with macrolide-resistant *S*. *pneumoniae* [17, 18]. Our previous study showed that more than 80% of *S*. *pneumoniae* isolated in municipal hospitals were non-susceptible to macrolides [19]. In addition to the appropriate prescription and consumption of antibiotics, new therapeutic agents need to be developed.

In this study, we investigated the effect of hinokitiol on bacterial loads and analyzed the suitable administration route of hinokitiol using a murine pneumococcal infection model. Furthermore, we also examined the effect of hinokitiol on inflammation in a severe pneumonia murine model.

## Materials & methods

### Bacterial strains

Antimicrobial-susceptible *S*. *pneumoniae* strain D39 (serotype 2) was purchased from the National Collection of Type Cultures (Salisbury, UK). Macrolide-resistant *S*. *pneumoniae* clinical strain NU4471 (serotype 19, azithromycin minimum inhibitory concentration (MIC) ≥ 1 mg/mL) was isolated from patients with respiratory tract infections [20]. All strains were grown in tryptic soy broth (Becton Dickinson, Franklin Lakes, NJ, USA) at 37°C as described previously [21].

### Animals and reagents

Male 8-10-week-old BALB/c mice were obtained from Nihon CLEA Tokyo, Japan. Mice were maintained under standard conditions in accordance with our institutional guidelines. All animal experiments were approved by the Institutional Animal Care and Use Committee of Niigata University (SA00223). Hinokitiol was purchased from FUJIFILM Wako Pure Chemical Industries (Osaka, Japan) and solubilized in phosphate- buffered saline (PBS) containing 10% ethanol. Thiazolyl blue tetrazolium bromide (MTT) was purchased from Sigma-Aldrich (St. Louis, MO, USA). The anesthetic mixture consisted of medetomidine hydrochloride (Domitol; Meiji Seika Pharma Co., Ltd., Tokyo, Japan), midazolam (Dormicum; Astellas Pharma Inc., Tokyo, Japan), and butorphanol (Vetorphale; Meiji Seika Pharma Co., Ltd.) mixed at 0.15, 2.0, and 2.5 mg/kg body weight with normal saline (Otsuka Pharmaceutical Factory, Inc. Tokyo, Japan) [22].

### Evaluation of systemic toxicity of hinokitiol *in vivo*

BALB/c mice were injected intraperitoneally once a day with 0, 10, 20, 30, 50, and 100 mg/kg hinokitiol for 7 days. Body weight was monitored every day. The change in body weight was considered an indicator of systemic toxicity of hinokitiol. The To evaluate the toxicity of intratracheally administered hinokitiol, mice were injected 500 μg/mL hinokitiol in 50 μL PBS.

### Evaluation of *in vitro* cytotoxicity of hinokitiol

Human alveolar epithelial cell line A549 (ATCC CCL-185) was obtained from RIKEN Cell Bank (Ibaraki, Japan). Cells were seeded in a 96-well plate (Becton Dickinson) and grown to 90% confluence in Dulbecco‘s Modified Eagle Medium (FUJIFILM Wako Pure Chemical Industries) supplemented with 10% fetal bovine serum (Japan Bio Serum, Hiroshima, Japan) and 100 U/mL penicillin and 100 μg/mL streptomycin (both FUJIFILM Wako Pure Chemical Industries) at 37°C in 5% CO_2_. A549 cells were treated with various concentrations (10–1000 μg/mL) of hinokitiol or 0.5% Triton X-100 for 3 h. MTT assays were performed to determine cell viability as described previously [23].

### Pneumonia mouse model with *S*. *pneumoniae* and hinokitiol medication

Mice (*n* = 8 per group) were anesthetized with a mixture of medetomidine hydrochloride, midazolam, and butorphanol and were intratracheally infected with *S*. *pneumoniae* strain D39 or strain NU4471 [1.0 × 10^9^ colony forming units (CFU) in 50 μL PBS] using the MicroSprayer Aerosolizer (Penn-Century Inc., Philadelphia, PA, USA). Uninfected control mice (*n* = 5 per group) were intratracheally injected with 50 μL PBS (Uninfected-PBS group) or 500 μg/mL hinokitiol (Uninfected-hinokitiol group). At 1 h after infection, all infected mice were anesthetized again and 500 μg/mL hinokitiol (infected-hinokitiol group) was administered intratracheally to infected mice, while control mice were administered 50 μL PBS (infected-PBS group). To evaluate the efficacy of repeated hinokitiol administration in a pneumonia mouse model, all mice were intratracheally infected with *S*. *pneumoniae* strain NU4471 (3.0 × 10^8^ CFU/mouse). After 1 h, 50 μL PBS was injected into the respiratory tract (Untreated group; *n* = 5), followed by intratracheal injection of PBS every 24 h. In the hinokitiol injection group, all mice were initially intratracheally administered with hinokitiol 1 h after infection. The single administration group (*n* = 5) was injected with PBS after initial hinokitiol administration at 24 h intervals. In the double administration group (*n* = 5), the second administration of hinokitiol was performed at 24 h after infection, and PBS was administrated after 48 h from infection. In the three-dose group (*n* = 5), hinokitiol was intratracheally administered every 24 h after the first administration. All mice were sacrificed 72 h after from pneumococcal infection.

### Bacteriological and pathological evaluation in mice lungs

At 18 h after infection, BALF and serum samples were collected from five mice. Lung tissue samples were collected from three mice. BALF samples were plated onto blood-agar plates and cultured aerobically to count CFU. To determine the concentration of pneumococcal DNA in murine serum, DNA was isolated from 50 μL of serum obtained from the infected mice using QIAamp Spin Columns (QIAGEN, Hilden, Germany). Then, absolute quantification by real-time PCR was performed using the StepOnePlus real-time PCR system via the SYBR Green detection protocol (Thermo Fisher Scientific, Waltham, MA, USA). The primers used for real-time PCR targeted a fragment of the pneumolysin-encoding gene of *S*. *pneumoniae* [24]. The forward primer sequence was 5′-AGCGATAGCTTTCTCCAAGTGG-3′ and the reverse primer sequence was 5′-CTTAGCCAACAAATCGTTTACCG-3′.

For pathological examinations, lung tissue samples were fixed with 4% paraformaldehyde (FUJIFILM Wako Pure Chemical Industries). Paraffin-embedded sections were made by Biopathology Institute Co., Ltd (Oita, Japan). Lung sections were stained with hematoxylin and eosin (Sakura Finetek Japan Co., Ltd., Tokyo, Japan). They were observed under a BIOREVO BZ-9000 microscope (Keyence, Osaka, Japan). To detect neutrophil elastase (NE) in lung tissue, paraffin-embedded sections were treated with rabbit anti-mouse NE antibody (Abcam, Cambridge, UK) in blocking solution (Thermo Fisher Scientific). After overnight incubation at 4°C, the secondary AlexaFluor 488-conjugated goat anti-rabbit IgG antibody (Thermo Fisher Scientific) in blocking buffer was added, followed by a 2 h incubation in the dark. The samples were then washed with PBS and mounted with nuclear staining mounting medium (ProLong^TM^ Diamond Antifade Mountant with DAPI; Thermo Fisher Scientific). Finally, samples were observed with a confocal laser-scanning microscope (Carl Zeiss, Jena, Germany).

### Flow cytometry analysis

The number of neutrophils in BALF was counted by flow cytometry. Prior to staining cells in BALF, their surfaces were blocked with anti-CD16/32 antibody (Thermo Fisher Scientific) at 4°C for 5 min [25]. Then, cells were stained with anti-CD11b-allophycocyanin and Ly-6G-phycoerythrin antibody (Thermo Fisher Scientific) in PBS containing 1% bovine serum albumin (Sigma-Aldrich) at 4°C for 40 min in the dark [26]. Samples were washed and fixed with 4% paraformaldehyde in PBS. Flow cytometry analysis was performed on a NovoCyte flow cytometer with NovoExpress software (ACEA Biosciences, San Diego, CA, USA) [26]. The cells were gated by their forward and side scatter. Neutrophils were identified by the expression of both Ly-6G and CD11b [26].

### Neutrophil elastase activity assay

NE activity in BALF was determined using the specific substrate *N*-methoxysuccinyl Ala-Ala-Pro-Val p-nitroanilide (Merck Millipore, Billerica, MA, USA) as described previously [27]. Briefly, samples were incubated in 0.1 M Tris-HCl (pH 8.0) containing 0.5 M NaCl and 1 mM substrate at 37°C for 24 h, after which absorbance at 405 nm was measured.

### Cytokine and chemokine analysis in BALF and serum

For cytokine and chemokine measurements, BALF samples were centrifuged at 2000 × *g* at 4°C for 15 min. Then, enzyme-linked immunosorbent assay was performed to determine the concentrations of (i) C-X-C motif chemokine ligand l (CXCL-1; ProteinTech Group, Chicago, IL, USA), (ii) interleukin (IL)-1β, IL-6, and tumor necrosis factor (TNF) (BioLegend Inc., San Diego, CA, USA) in BALF and serum according to the manufacturers’ instructions.

### Statistical analysis

Data were analyzed statistically by analysis of variance with Tukey’s multiple-comparison test. Where appropriate (comparison of two groups only), unpaired *t*-tests were conducted. All statistical analyses were performed using GraphPad Prism Software version 8.03 (GraphPad Software, Inc., La Jolla, CA, USA). Values of *P* < 0.05 were considered significant.

## Results

### Systemic administration of hinokitiol does not ameliorate pneumococcal pneumonia in mice

We first evaluated the systemic toxicity of hinokitiol. BALB/c mice were intraperitoneally injected with hinokitiol once a day for 7 days as specified in the Supplementary Information. As shown in S1 Fig, administration of ≥20 mg/kg hinokitiol significantly decreased body weight 3 days after the first administration (*P* < 0.05). Hence, we lowered the maximum hinokitiol concentration for systemic administration in mice to 10 mg/kg. We used this dosage to test whether intraperitoneal administration of hinokitiol ameliorated pneumonia in a mouse intratracheal pneumococcal infection model. There was no significant decrease on pneumococcal CFU in the bronchoalveolar lavage fluid (BALF) (S2 Fig; *P* > 0.05) and little effect on the transcriptional levels of various cytokine genes, such as *Cxcl1*, *Il1b*, *Il6*, and *Tnf*, in lung tissue (S3 Fig; *P* > 0.05).

### Up to 500 μg/mL (833 μg/kg) of hinokitiol does not cause toxicity *in vitro* and *in vivo*

**Fig 1:**
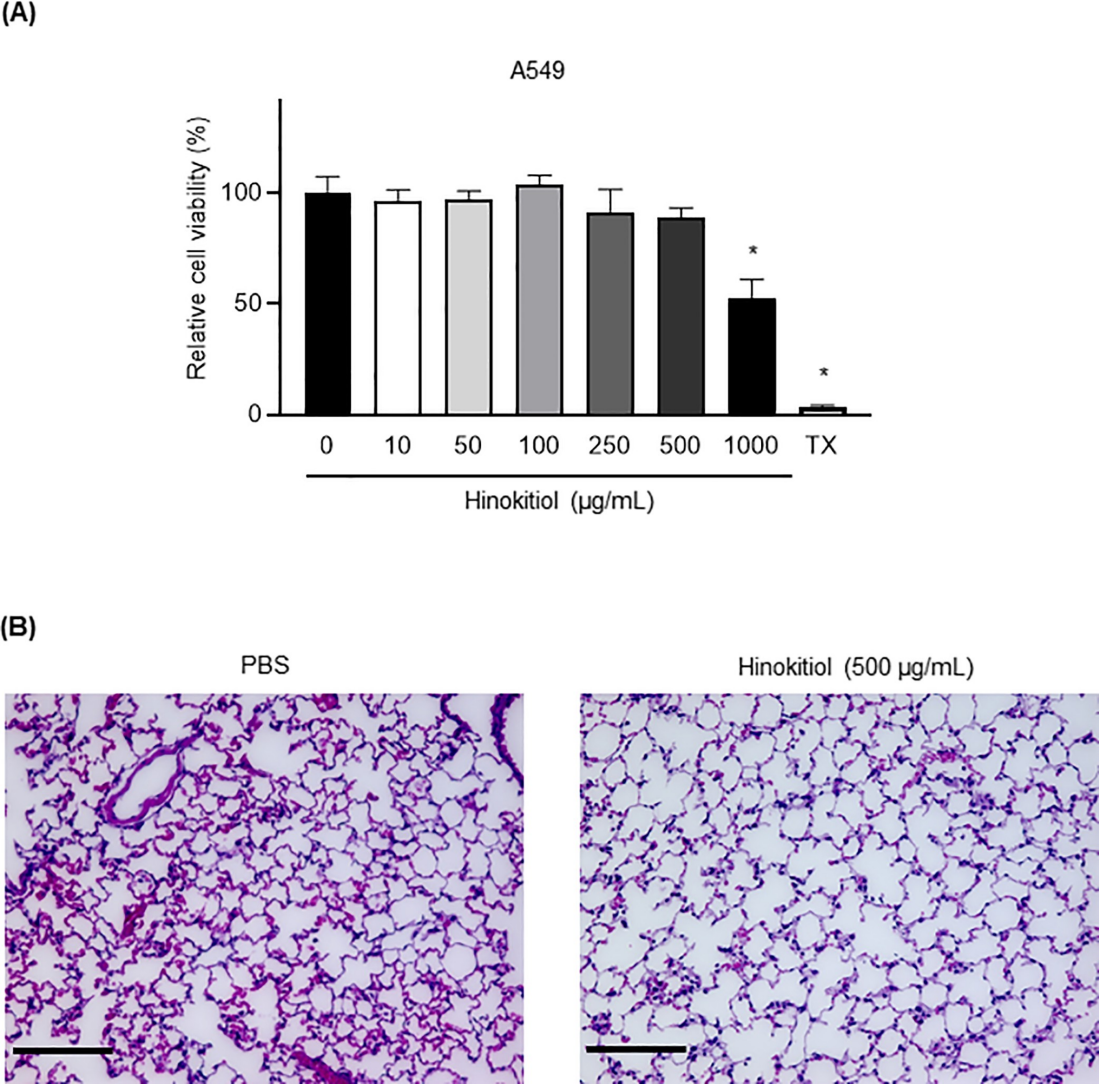
Cytotoxicity of hinokitiol. (A) Viability of A549 cells treated with various concentrations (10–1000 μg/mL) of hinokitiol or 0.5% Triton X-100 (TX) for 3 h and subjected to MTT assay. Data represent the mean ± SD of quintuplicate determinants and were evaluated using Dunnett’s multiple-comparisons test; *P < 0.05 compared with the untreated group. (B) Representative hematoxylin and eosin-stained lung tissue sections from mice injected with either PBS or hinokitiol (500 μg/mL). Magnification: 20×; scale bar: 50 μm.

The negative results from systemic administration of hinokitiol prompted us to test whether intratracheal administration of hinokitiol represented a better route for therapeutic intervention in pneumococcal pneumonia. Evaluation of hinokitiol cytotoxicity against A549 alveolar epithelial cells *in vitro* revealed no significant decrease in viability except at 1000 μg/mL, whereby cell viability was reduced by 54% (Fig 1A; *P* < 0.05). Furthermore, compared with PBS injection, intratracheal hinokitiol administration caused negligible morphological changes in lung tissues in mice (Fig 1B).

### Intratracheal injection of hinokitiol ameliorates pneumococcal pneumonia in mice

**Fig 2:**
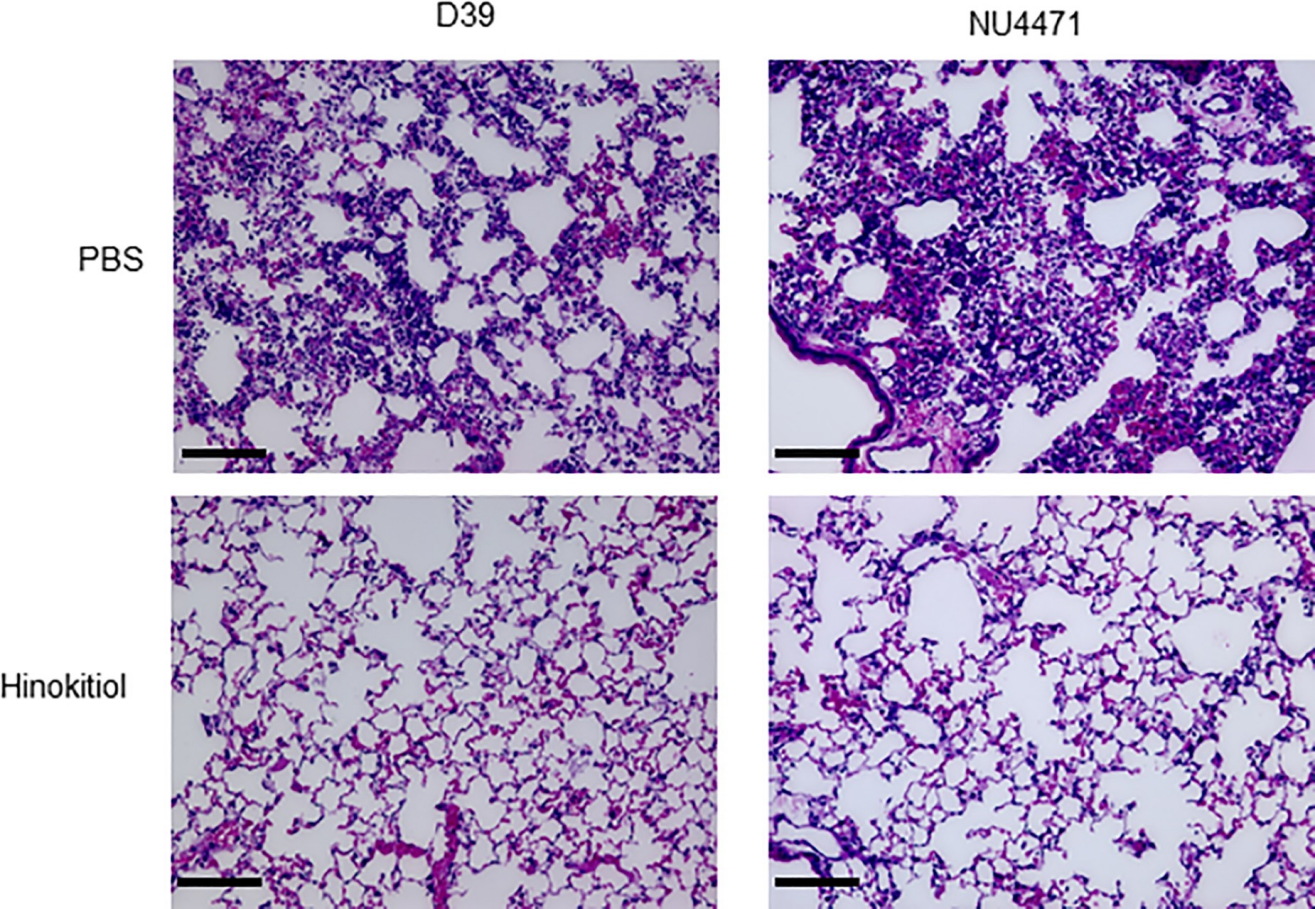
Hinokitiol injection suppresses inflammation in the lungs. Representative hematoxylin and eosin-stained lung tissue sections from 8-week-old BALB/c mice intratracheally infected with antimicrobial-susceptible *S*. *pneumoniae* strain D39 or macrolide-resistant strain NU4471 (1.0 × 10^9^ CFU in 50 μL PBS) and then injected intratracheally with PBS or hinokitiol (500 μg/mL) 1 h after infection. Magnification: 20×; scale bar: 50 μm.

**Fig 3:**
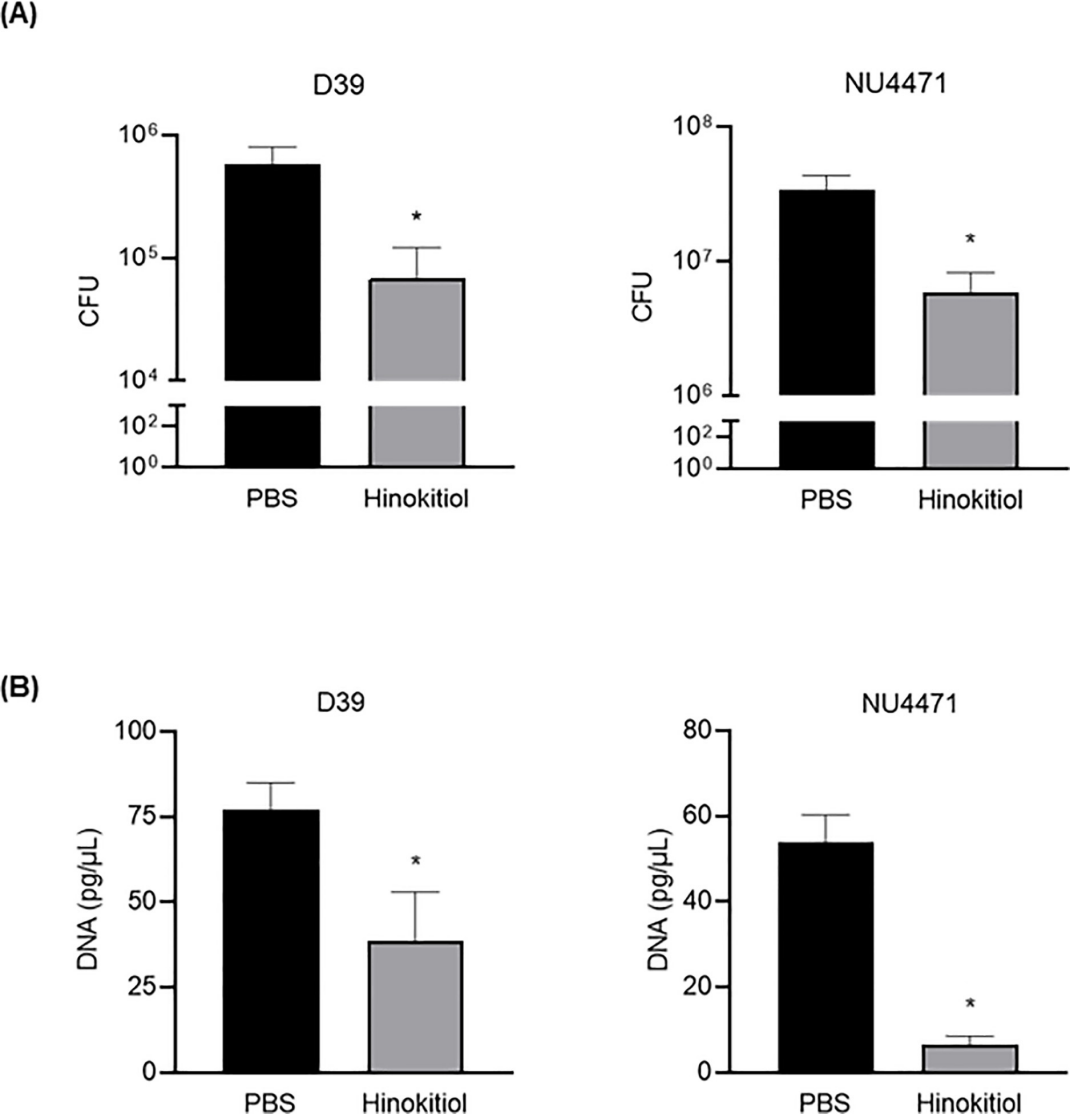
Intratracheal hinokitiol injection decreases bacterial load in BALF and serum. (A) CFU counts of antimicrobial-susceptible *S*. *pneumoniae* strain D39 or macrolide-resistant strain NU4471 in BALF samples plated on blood-agar plates and cultured aerobically. (B) Real-time PCR results showing *S*. *pneumoniae* DNA (*pneumolysin* gene) concentration in serum. Data represent the mean ± SEM (*n* = 5) and were analyzed using Student’s *t*-test; *P < 0.05.

Our previous *in vitro* study showed that 0.3 μg/mL hinokitiol exerted antibacterial effect against *S*. *pneumoniae* regardless of antimicrobial resistance [10]. Here, compared with PBS injection, intratracheal administration of 500 μg/mL hinokitiol in a pneumococcal pneumonia mouse model decreased inflammatory cell migration and prevented destruction of alveolar tissue (Fig 2). In addition, intratracheal administration of hinokitiol significantly decreased CFU in BALF of both antimicrobial-susceptible strain D39 and macrolide-resistant strain NU4471 (Fig 3A; *P* < 0.05). Because the severity of pneumococcal pneumonia is related to bacteremic pneumonia [28], next, we investigated whether hinokitiol treatment inhibited *S*. *pneumoniae*. Fig 3B shows that compared with PBS injection in mice, hinokitiol administration significantly decreased *S*. *pneumoniae* serum DNA concentration (*P* < 0.05).

### Intratracheal hinokitiol injection decreases neutrophil recruitment to the lungs of mice infected with *S*. *pneumoniae*

**Fig 4:**
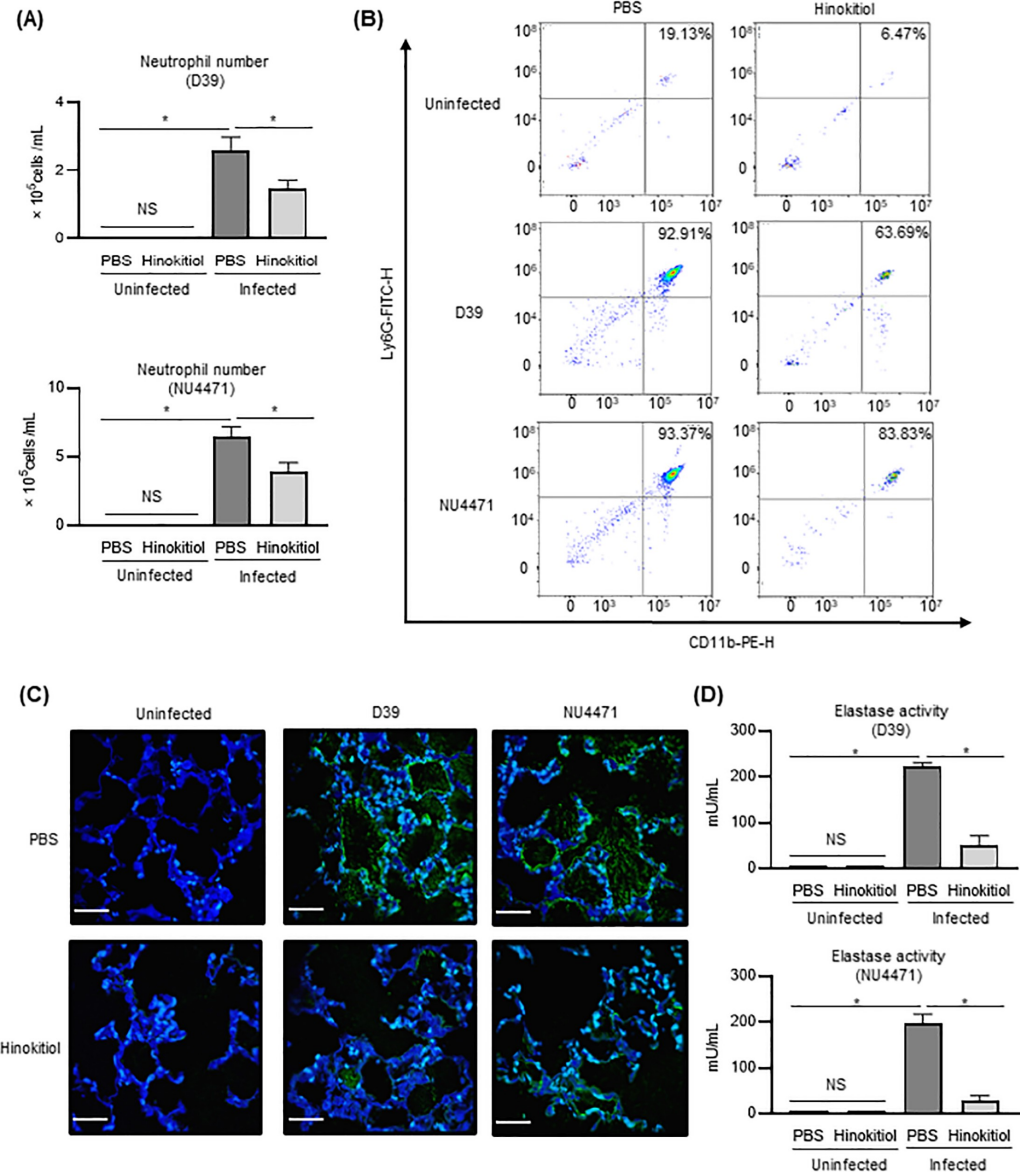
Neutrophil infiltration in mice lungs. (A) Number of neutrophils in BALF was determined by flow cytometry. (B) Representative FACS plots were chosen. (C) Representative fluorescence microscopy images of lungs from mice infected with *S*. *pneumoniae* strain D39 or strain NU4471 in the presence or absence of hinokitiol intratracheal injection: Neutrophil elastase (NE: green), DAPI (blue). Magnification: 40×; scale bar: 50 μm. (D) NE activity in BALF of mice infected with either antimicrobial-susceptible *S*. *pneumoniae* strain D39 or macrolide-resistant strain NU4471 and then treated with PBS or hinokitiol. Data represents the mean ± SEM (*n* = 5) and was evaluated using one-way analysis of variance with Tukey’s multiple-comparisons test. *Significantly different from the infected control group at *P* < 0.05. ND: not-detected, NS: not-significant; *P < 0.05.

Previous studies have shown that excessive activation of neutrophils causes the release of NE, which contributes to severe lung injury [28]. Therefore, controlling the excessive accumulation of neutrophils may be an essential therapeutic strategy to combat severe pneumonia [28, 29]. Fig 4A shows the number of Ly6G^+^, CD11b^+^ cells, which considered neutrophils in the pro-inflammatory cells in BALF. No significant increase in neutrophils was observed between the uninfected-PBS group and the uninfected-hinokitiol group. Meanwhile, the number of neutrophils was significantly decreased in the BALF of the infected-hinokitiol group compared with the infected-PBS group (*P* < 0.05). Fig 4B shows representative flow cytometric panels for one of the five mice in each group. The proportion of neutrophils was decreased in the hinokitiol-administered group (D39: 63.69%, NU4471: 83.83%) compared to the non-administered group (D39: 92.91%, NU4471: 93.37%). Furthermore, hinokitiol treatment down-regulated the NE level in mouse lung compared with the untreated mouse in the infected group (Fig 4C). Similarly, NE activity in BALF was significantly decreased in the hinokitiol administrated group which infected with pneumococcus compared to PBS injected group (Fig 4D; *P* < 0.05).

### Hinokitiol treatment lowers the levels of inflammatory cytokines in BALF and serum

**Fig 5:**
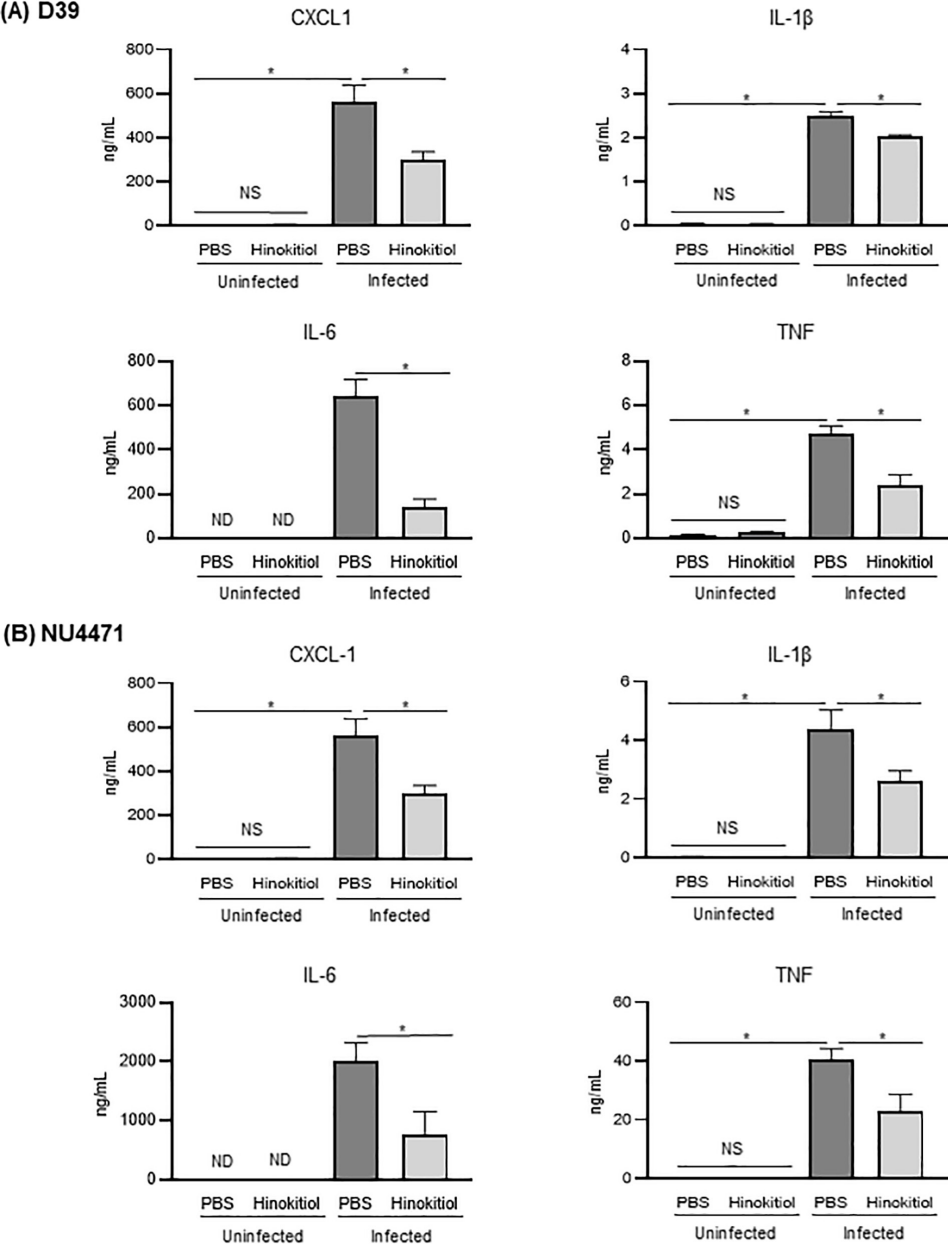
Chemokine and cytokine concentrations in mouse BALF. Concentration of CXCL-1, IL-1β, IL-6, and TNF in BALF from mice infected with (A) *S*. *pneumoniae* strain D39 or (B) *S*. *pneumoniae* strain NU4471 and then treated with either PBS or hinokitiol, as determined by enzyme-linked immunosorbent assays. Unchallenged naive mice were administered PBS or hinokitiol only. Data represent the mean ± SEM (*n* = 5) and were evaluated using one-way analysis of variance with Tukey’s multiple-comparisons test. *Significantly different from the infected control group at *P* < 0.05. ND: not-detected, NS: not-significant.

**Fig 6:**
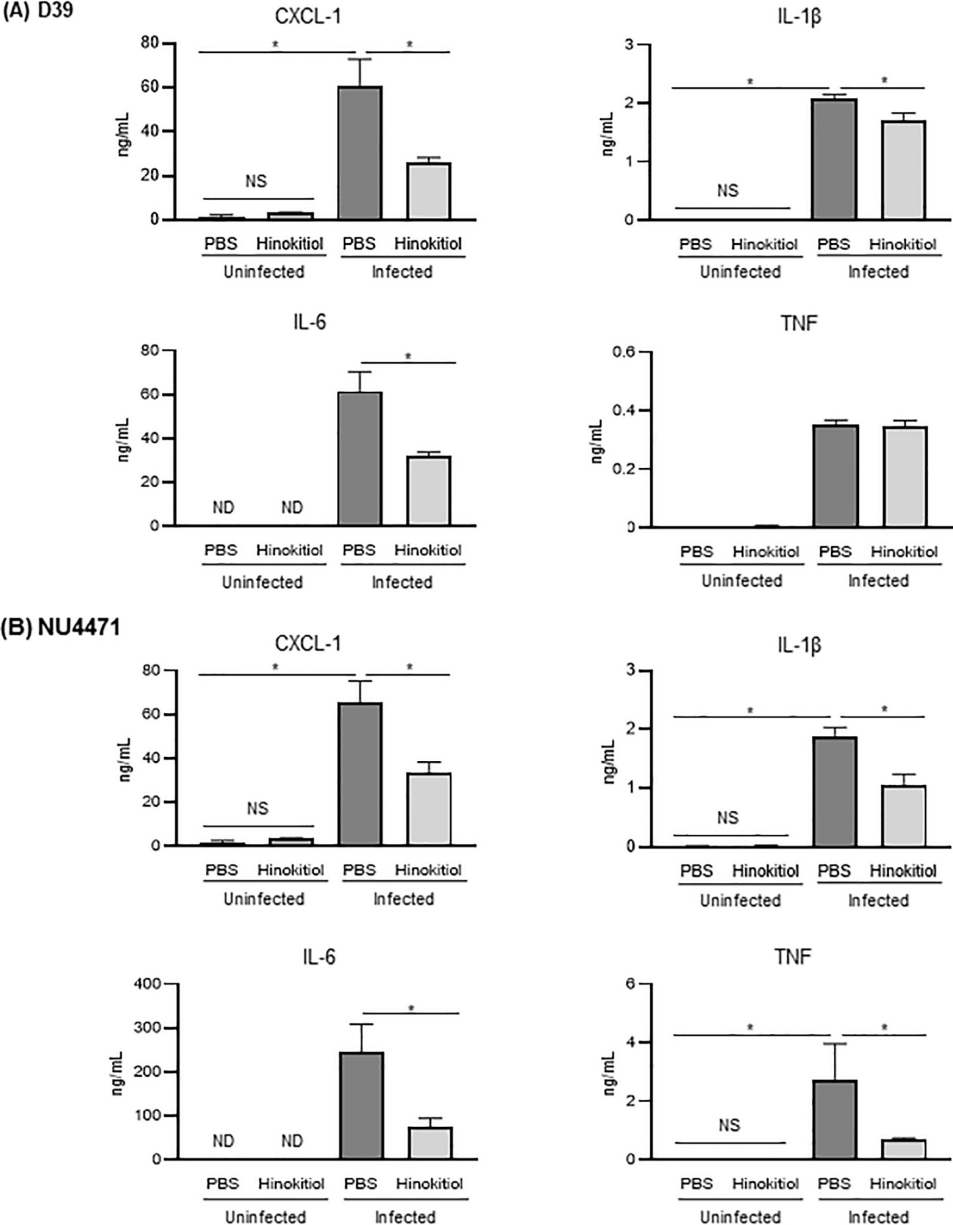
Chemokine and cytokine concentrations in mouse serum. Concentration of CXCL-1, IL-1β, IL-6, and TNF in BALF from mice infected with (A) *S*. *pneumoniae* strain D39 or (B) *S*. *pneumoniae* strain NU4471 and then treated with either PBS or hinokitiol, as determined by enzyme-linked immunosorbent assays. Unchallenged naive mice were administered PBS or hinokitiol only. Data represent the mean ± SEM (*n* = 5) and were evaluated using one-way analysis of variance with Tukey’s multiple-comparisons test. *Significantly different from the infected control group at *P* < 0.05. ND: not-detected, NS: not-significant

Excessive production of inflammatory cytokines or chemokines is a hallmark of tissue injury and severe pneumonia [30, 31]. The concentration of CXCL-1, IL-1β, IL-6, and TNF was significantly lower in the BALF from the infected-hinokitiol group compared with that from the infected-PBS group (Fig 5; *P* < 0.05). Serum IL-6 and TNF levels are associated with mortality of patients with pneumonia [32]. Our previous study revealed that pneumococcal bacteremia induced an increase in serum cytokine levels [33]. Here, the concentration of CXCL-1, IL-1β, and IL-6, but not TNF, was significantly decreased in the serum of the infected-hinokitiol group compared with that in the infected-PBS group (Fig 6; *P* < 0.05). Meanwhile the chemokine and cytokines concentrations in BALF and serum of uninfected control groups were either below (IL-6) or just above the limit of detection (CXCL-1, IL-1β and TNF). Taken together, these findings suggested that the antibacterial action of hinokitiol in the lungs of pneumonia mice reduced the production of cytokines or chemokines in BALF and serum.

### Repeated hinokitiol injection ameliorates pneumococcal pneumonia in mice

**Fig 7:**
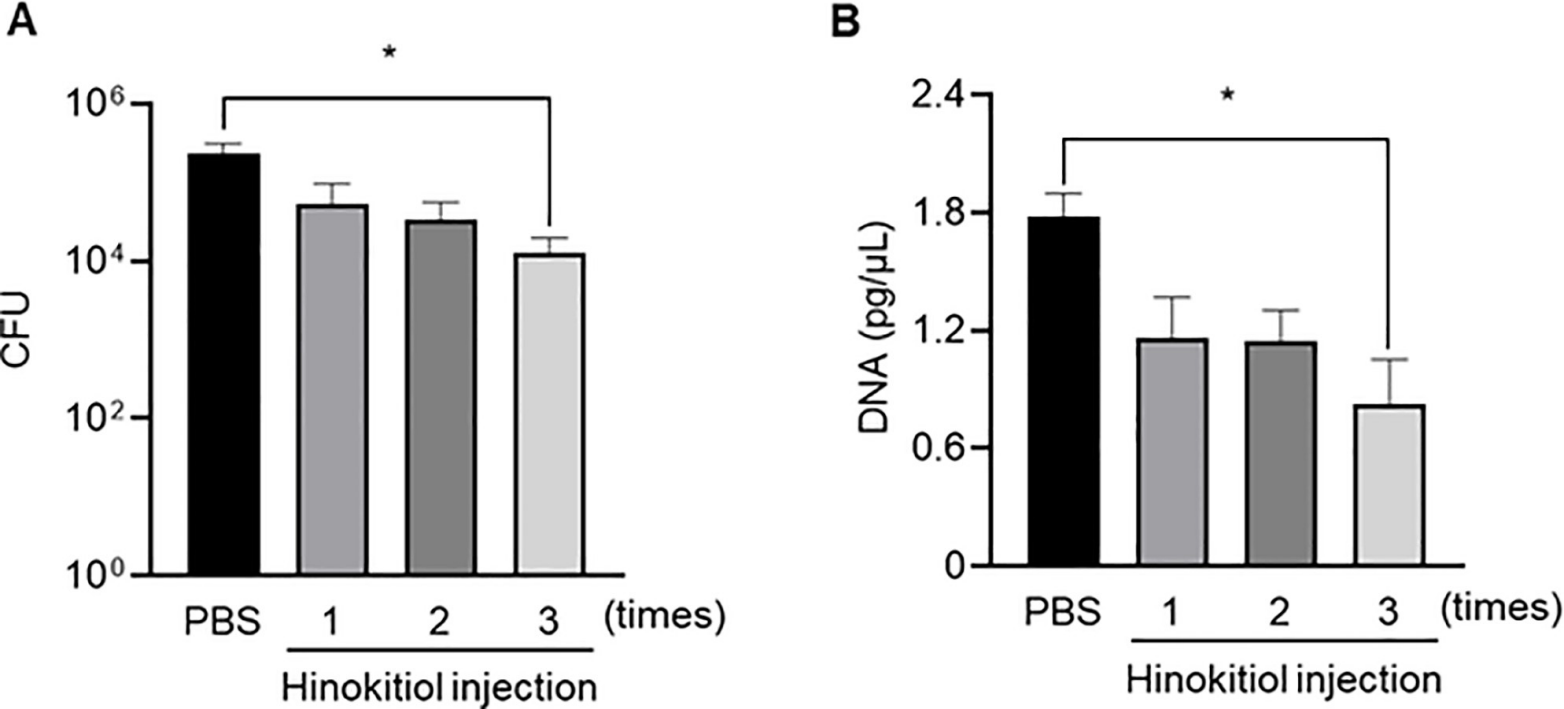
Effect of repeated hinokitiol injection on bacterial load in the lungs and serum. Mice were intratracheally infected with *S*. *pneumoniae* NU4471 (3.0 × 10^8^ CFU in 50 μL PBS). Hinokitiol (500 μg/mL) or only PBS was injected intratracheally once, twice, or three times at 24 h intervals. BALF and serum samples were collected 72 h after infection. (A) CFU counts in BALF samples plated on blood-agar plates and cultured aerobically. (B) Real-time PCR results showing *S*. *pneumoniae* DNA (*ply*) concentration in serum. Data represent the mean ± SEM (*n* = 5) and were analyzed using ANOVA with Turkey’s multiple comparisons test; *P < 0.05.

The above results suggested that intratracheal hinokitiol administration was effective in inhibiting *S*. *pneumoniae* in mouse lung. However, in clinical settings, antimicrobial agents are administrated several times for the treatment of pneumococcal pneumonia, and thus, we next sought to investigate whether repeated hinokitiol injection decreased the bacterial load in BALF and serum. The experimental schedule was presented in S4 Fig. The experimental mice that were injected three times with hinokitiol over three days, had significantly lower BALF and *S*. *pneumoniae* DNA levels at 72 hours post-infection compared with the untreated group (Fig 7; *P* < 0.05). Meanwhile, no significant differences were observed in BALF and serum bacterial loads at 72 hours post-infection in the group between the untreated group and the groups which administrated hinokitiol once or twice. These results suggest that repeated administration of hinokitiol is required for the effective treatment of pneumococcal pneumonia.

## Discussion

The present study showed that intratracheal administration of hinokitiol decreased the bacterial load in a mouse pneumonia model regardless of macrolide resistance of *S*. *pneumoniae*. As a result, the production of inflammatory cytokines and release of NE also decreased, eventually preventing the disruption of lung tissue. These results suggest that the antibacterial activity of hinokitiol has potential as a treatment against pneumococcal pneumonia.

An important aspect when selecting a drug is to evaluate and compare its therapeutic index [34]. Although hinokitiol exhibits antibacterial effect against various pathogenic organisms [10], dosages above 12.7 and 14.8 mg/kg/day in male and female rats, respectively, have been shown to produce systemic side effects such as chronic toxicity [35]. Based on our observations and these values, intraperitoneal injection of 10 mg/kg hinokitiol would be the maximum concentration for systemic administration in mice. However, this amount did not decrease bacterial load in our mouse pneumonia model, suggesting that this concentration is inadequate to achieve MIC against *S*. *pneumoniae* in the lungs.

Topical administration of antibacterial agents has been successfully evaluated in the clinic for the prevention as well as therapeutic treatment of invasive pulmonary infections. Our *in vitro* findings indicate that hinokitiol exhibited little cytotoxicity against pulmonary alveolar epithelial cells. Aminoglycoside formulations for inhalation were developed to maximize drug delivery to the respiratory tract and minimize toxicity [36–38]. Opting for an alternative route such as intratracheal administration of hinokitiol achieved the MIC against *S*. *pneumoniae*, reducing disruption of lung tissue and suppressing bacteremia without systemic side effects.

Intratracheal administration demonstrated the efficacy of hinokitiol in a mouse pneumonia model; however, this may not necessarily indicate successful and complete treatment of pneumonia. Clinically, treatment of infectious diseases requires thorough bactericidal effect until the infectious inflammation is resolved [39, 40]. Therefore, a repeated dose of antibiotics is often prescribed to maintain an adequate concentration of the drug at the site of infection [41–43]. Our repeated administration experiment showed that although single or two-time hinokitiol administration did not significantly lower the bacterial load 4 days after infection, repeating the procedure three times successfully decreased the bacterial load in the mouse pneumonia model. This finding suggests that hinokitiol administrated intratracheally does not exhibit antibacterial activity for an extended period and repeated administration may be required to treat pneumococcal pneumonia. Effective use of hinokitiol will demand further research to optimize the administration interval and treatment period.

Antimicrobial resistance is a global problem [44]; one way to deal with it is by developing alternative antimicrobials [45], including from plant essential oils [46]. Hinokitiol has been shown to inhibit the growth of antimicrobial-resistant organisms *in vitro*, such as methicillin-resistant *Staphylococcus aureus* and multidrug-resistant *S*. *pneumoniae* [10, 27, 47]. In this study, intratracheal administration of hinokitiol suppressed bacterial pneumonia induced by macrolide-resistant pneumococci. Thus, hinokitiol could represent a new weapon against the growing class of macrolide-resistant *S*. *pneumoniae*.

Neutrophils are rapidly recruited to the lungs of pneumonia patients [48]. Previous studies have shown that *S*. *pneumoniae* induced the release of NE from neutrophils, leading to the disruption of alveolar epithelial cells [28]. Moreover, several animal studies have reported that the inhibition of NE activity suppressed lung injury in pneumococcal pneumonia [49, 50]. In this study, intratracheal hinokitiol administration reduced neutrophil infiltration and elastase release in the lungs of pneumonia mice, consequently diminishing lung injury and pneumococcal DNA detection in serum.

Excessive production of inflammatory mediators induces a severe systemic response in pneumonia [51]. Although antibacterial treatment is an important approach against pneumonia, regulating excessive inflammation is equally essential [52, 53]. Hinokitiol was previously shown to reduce the transcription of inflammatory cytokines from epithelial cells *in vitro* [6, 54]. In this study, intratracheal administration of hinokitiol decreased the concentrations of cytokines and chemokines in BALF and serum. These findings suggest that the anti-inflammatory property of hinokitiol, in addition to its antibacterial activity, might ameliorate the severity of pneumonia by downregulating the production of inflammatory mediators.

Certain limitaitions are noted in the current study. First of all, the administration of hinokitiol 1 h after bacterial inoculation is not representative of the clinical scenario, where antibiotics and other therapies are often initiated days after infection onset. Additionally, most mouse experiments were only evaluated at a single time point in this study as obtaining BALF and lung tissues requires the mice to be sacrificed. Finally, the study does not include any conventional antibiotic comparators. The results of multiple doses indicate the limit of the antibacterial effect of hinokitiol and are expected to play a role as an adjunct to other antibacterial drugs. Therefore, further research is needed to apply hinokitiol to the treatment of pneumonia, including hinokitiol alone as well as in combination with existing antibacterial drugs and hinokitiol.

In conclusion, the present study showed that intratracheal hinokitiol administration suppresses acute pneumococcal pneumonia *in vivo*, regardless of whether bacteria are susceptible to macrolides. In case of severe pneumonia caused by antimicrobial-resistant pneumococci, a systemic dose of existing antimicrobials alone may not be successful [55, 56]. Adjunctive use of hinokitiol may facilitate the treatment of pneumococcal pneumonia and reduce the use of existing antimicrobials.

## Supporting information

S1 Fig Changes in body weight in mice intraperitoneally injected with hinokitiol. Male 8-week-old BALB/c mice were intraperitoneally injected once a day with 0, 10, 20, 30, 50, and 100 mg/ kg hinokitiol in PBS containing 10% ethanol buffer for 7 days. Body weight was monitored every day. Data represent the mean ± SEM (*n* = 4) and were analyzed using two-way ANOVA with Dunnett's multiple comparison test; *P < 0.05.
(TIF)

S2 Fig Intraperitoneal hinokitiol injection does not decrease the number of live bacteria in BALF. Male 8-week-old BALB/c mice were intratracheally infected with *S*. *pneumoniae* strain D39 (1.0 × 10^9^ CFU in 50 μL PBS) and, 1 h after infection, they (*n* = 5) were intraperitoneally injected with hinokitiol (10 mg/kg, *n* = 5) or PBS (containing 10% ethanol, *n* = 5). After 18 h, BALF samples were plated on blood-agar plates and cultured aerobically to count CFU. Data represent the mean ± SEM (*n* = 5) and were analyzed using Student’s *t*-test; *P < 0.05.
(TIF)

S3 Fig Hinokitiol does not affect the transcription of chemokine and cytokine genes in mice. Real-time PCR was performed to quantify the transcription of *Cxcl1*, *Il1b*, *Il6*, and *Tnf* in mouse lung tissue. Total RNA was extracted from mouse lung tissue using TRI reagent (Molecular Research Center, Inc., Cincinnati, OH, USA), and quality was assessed spectrophotometrically at 260 and 280 nm. The RNA was reverse-transcribed using SuperScript VILO Master Mix (Thermo Fisher Scientific, Waltham, MA, USA) and the cDNA was quantified using the StepOnePlus real-time PCR system according to the manufacturer’s protocol. Values were normalized to those of GAPDH mRNA and are presented as fold change relative to the mRNA transcript levels of the control group. Data represent the mean ± SEM (*n* = 5) and were analyzed using Student’s *t*-test; *P < 0.05.
(TIF)

S4 Fig Experimental schedule of repeated hinokitiol injection. All mice were intratracheally infected with *S*. *pneumoniae* strain NU4471 (3.0 × 10^8^ CFU/mouse). PBS injection or hinokitiol (500 μg/mL in PBS) injection via the tracheal route was started after 1 h from infection. PBS or hinokitiol injection into the air tract was performed at 24 h intervals. All mice were sacrificed and samples were collected after 72 h from infection.
(TIF)
