# Supplementary figures and images for: Treatment of severe pneumonia by hinokitiol in a murine antimicrobial-resistant pneumococcal pneumonia model
Source: PLoS One. 2020 Oct 15;15(10):e0240329. doi: 10.1371/journal.pone.0240329 (PMC7561173; doi:10.1371/journal.pone.0240329)

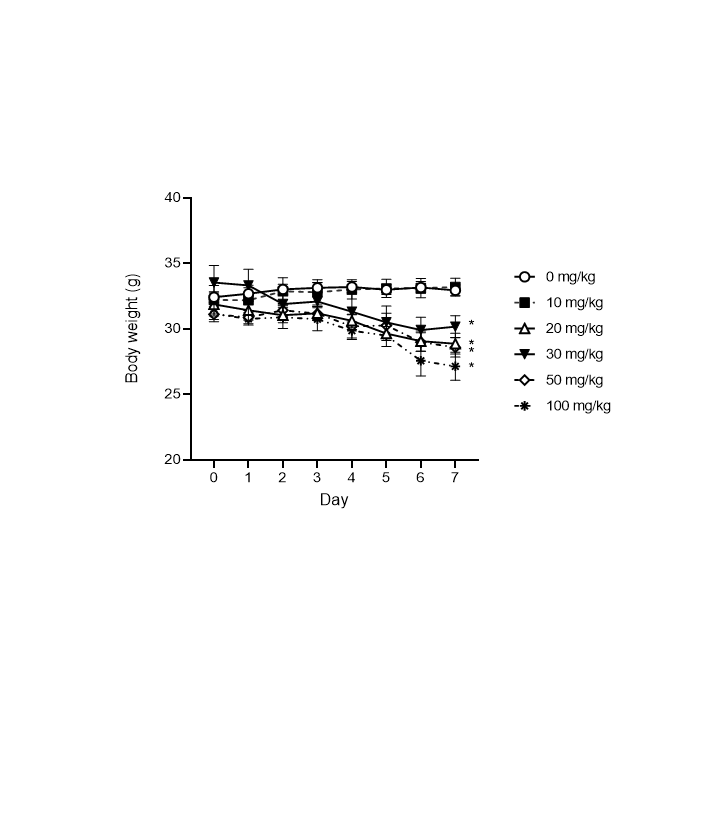

Supplement: S1 Fig — Male 8-week-old BALB/c mice were intraperitoneally injected once a day with 0, 10, 20, 30, 50, and 100 mg/ kg hinokitiol in PBS containing 10% ethanol buffer for 7 days. Body weight was monitored every day. Data represent the mean ± SEM (n = 4) and were analyzed using two-way ANOVA with Dunnett's multiple comparison test; *P < 0.05. (TIF) [file pone.0240329.s001.tif]

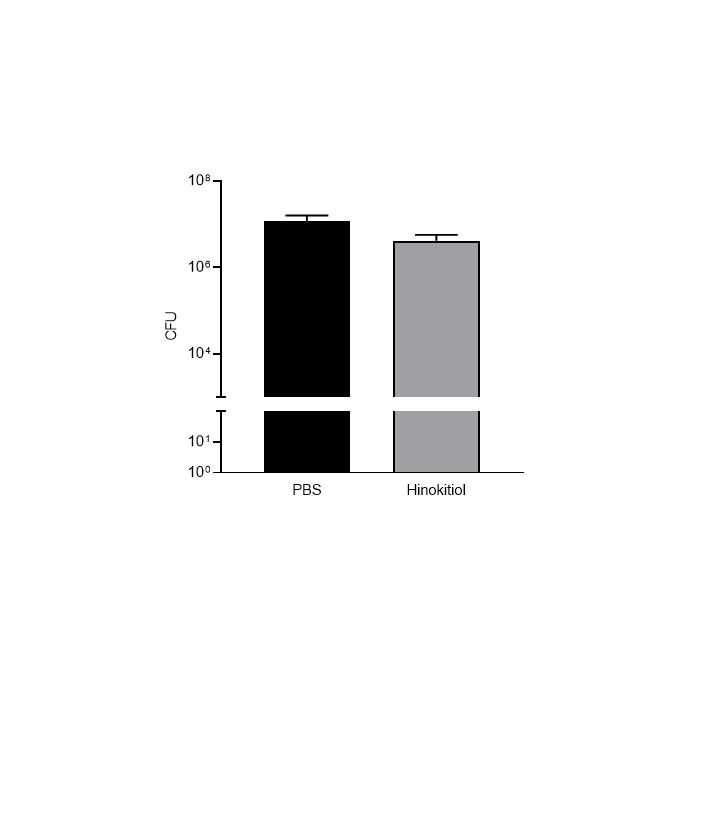

Supplement: S2 Fig — Male 8-week-old BALB/c mice were intratracheally infected with S. pneumoniae strain D39 (1.0 × 10^9^ CFU in 50 μL PBS) and, 1 h after infection, they (n = 5) were intraperitoneally injected with hinokitiol (10 mg/kg, n = 5) or PBS (containing 10% ethanol, n = 5). After 18 h, BALF samples were plated on blood-agar plates and cultured aerobically to count CFU. Data represent the mean ± SEM (n = 5) and were analyzed using Student’s t-test; *P < 0.05. (TIF) [file pone.0240329.s002.tif]

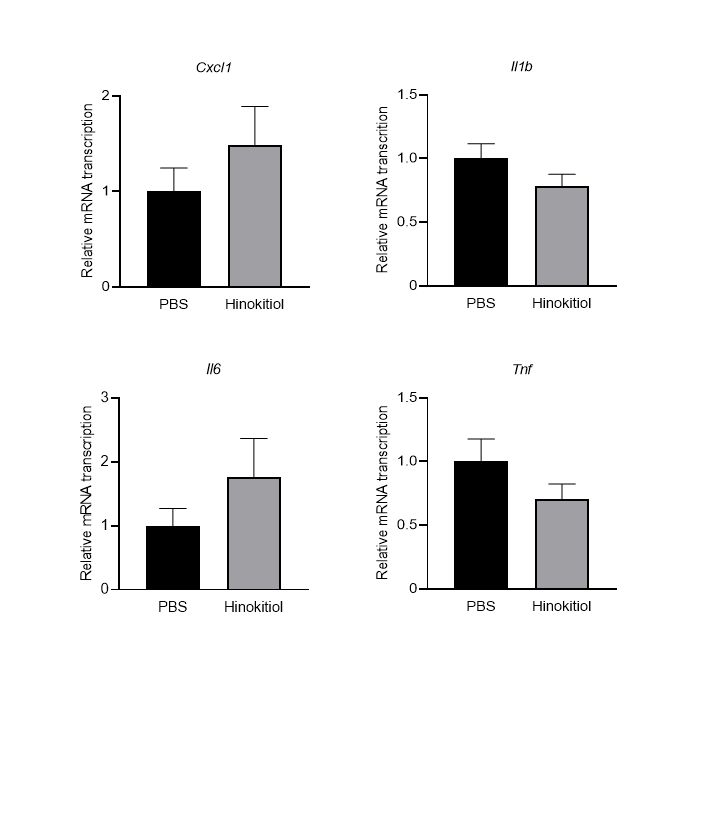

Supplement: S3 Fig — Real-time PCR was performed to quantify the transcription of Cxcl1, Il1b, Il6, and Tnf in mouse lung tissue. Total RNA was extracted from mouse lung tissue using TRI reagent (Molecular Research Center, Inc., Cincinnati, OH, USA), and quality was assessed spectrophotometrically at 260 and 280 nm. The RNA was reverse-transcribed using SuperScript VILO Master Mix (Thermo Fisher Scientific, Waltham, MA, USA) and the cDNA was quantified using the StepOnePlus real-time PCR system according to the manufacturer’s protocol. Values were normalized to those of GAPDH mRNA and are presented as fold change relative to the mRNA transcript levels of the control group. Data represent the mean ± SEM (n = 5) and were analyzed using Student’s t-test; *P < 0.05. (TIF) [file pone.0240329.s003.tif]

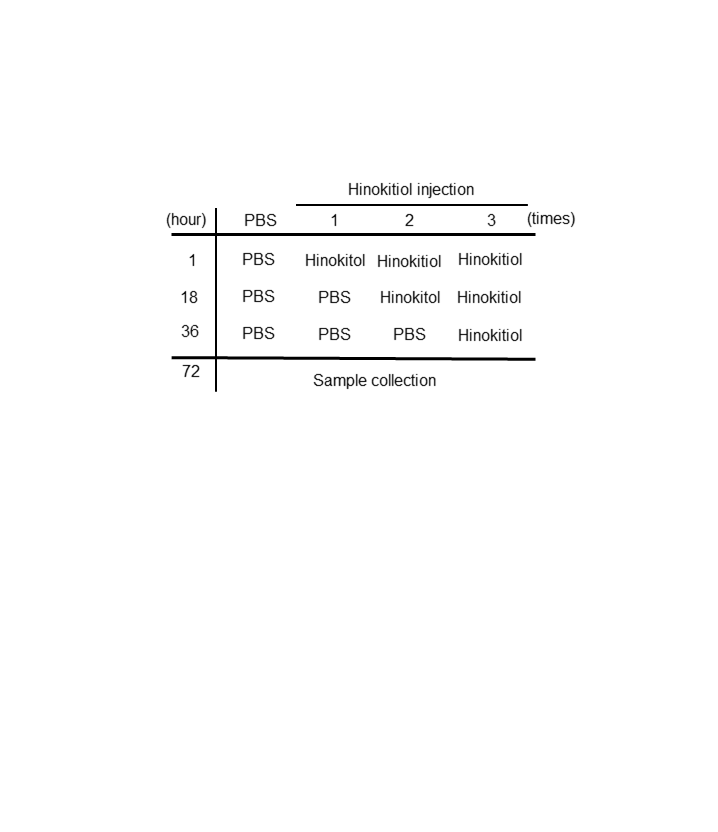

Supplement: S4 Fig — All mice were intratracheally infected with S. pneumoniae strain NU4471 (3.0 × 10^8^ CFU/mouse). PBS injection or hinokitiol (500 μg/mL in PBS) injection via the tracheal route was started after 1 h from infection. PBS or hinokitiol injection into the air tract was performed at 24 h intervals. All mice were sacrificed and samples were collected after 72 h from infection. (TIF) [file pone.0240329.s004.tif]
